# Gram-Negative Bacterial Infection in Thigh Abscess Can Migrate to Distant Burn Depending on Burn Depth

**DOI:** 10.1155/2012/567140

**Published:** 2012-07-26

**Authors:** Victoria Hamrahi, Michael R. Hamblin, Walter Jung, John B. Benjamin, Kasie W. Paul, Alan J. Fischman, Ronald G. Tompkins, Edward A. Carter

**Affiliations:** ^1^Department of Bacteriology, Shriners Hospitals for Children, Boston, MA 02114, USA; ^2^Department of Pediatrics, Harvard Medical School, Boston, MA 02115, USA; ^3^Wellman Center for Photomedicine, Massachusetts General Hospital, Boston, MA 02114, USA; ^4^Department of Dermatology, Harvard Medical School, Boston, MA 02115, USA; ^5^Department of Dermatology, Harvard-MIT Division of Health Sciences and Technology, Cambridge, MA 02139, USA; ^6^Pediatric Department, Massachusetts General Hospital, Boston, MA 02115, USA; ^7^Department of Surgery, Harvard Medical School, Boston, MA 02115, USA; ^8^Trauma, Emergengcy Surgery and Surgical Critical Care Service, Massachusetts General Hospital, Boston, MA 02114, USA

## Abstract

Sepsis remains the major cause of death in patients with major burn injuries. In the present investigation we evaluated the interaction between burn injuries of varying severity and preexisting distant infection. We used Gram-negative bacteria (*Pseudomonas aeruginosa* and *Proteus mirabilis*) that were genetically engineered to be bioluminescent, which allowed for noninvasive, sequential optical imaging of the extent and severity of the infection. The bioluminescent bacteria migrated from subcutaneous abscesses in the leg to distant burn wounds on the back depending on the severity of the burn injury, and this migration led to increased mortality of the mice. Treatment with ciprofloxacin, injected either in the leg with the bacterial infection or into the burn eschar, prevented this colonization of the wound and decreased mortality. The present data suggest that burn wounds can readily become colonized by infections distant from the wound itself.

## 1. Introduction

Infection is the most common and most serious complication of major burn injuries and is related to burn size and severity of the injury [1]. Currently, sepsis accounts for 50–60% of deaths in burn patients despite major improvements in antimicrobial therapies. Microbial colonization of the open burn wounds is usually established by the end of the first week after injury [1] despite the use of antimicrobial agents. If the bacterial density overwhelms the immune defenses of the host, invasive burn sepsis may ensue. The burn wound becomes colonized by bacteria in part because of the loss of the skin barrier function, and in part because the burn wound has few or no blood vessels which prevents the antimicrobial action of blood-borne cells from the host immune system.

Burn injury leads to suppression of nearly all aspects of the immune response [2]. Postburn serum levels of immunoglobulins, fibronectin, and complement are reduced, and there is a diminished capacity for opsonization of bacteria. Chemotaxis, phagocytosis, and killing functions of neutrophils, monocytes, and macrophages are impaired. Granulocytopenia is common following burn injury. The cellular immune response is impaired, as evidenced by delayed allograft rejection, anergy to common antigens, impaired lymphocyte mitogenesis, and altered mixed lymphocyte responsiveness. Burn injury results in reductions in interleukin-2 (Il-2) production, T-cell and NK cell cytotoxicity, and helper- to- suppressor T-cell ratio (HSR).

Clinical findings clearly suggest that burn size profoundly impacts patient immune status and survival of burn patients [3–6]. The depth of the burn wound injury has also been shown to affect permeability of the burned skin [7] and the content of natural antimicrobial peptides, which are expressed everywhere including the deeper portions of the skin [8]. In the present study we used bioluminescent bacteria to determine if an abscess caused by these microorganisms in a location (thigh) distant from and distal to the burn wound on the dorsum could result in colonization of the burn eschar. Our results suggest that the burn eschar becomes colonized from the bacteria injected in the thigh in relation to the depth of the burn injury. This increased colonization of the burn eschar by the bacteria injected in the thigh was associated with increased mortality, which could be completely prevented by the antibiotic ciprofloxacin.

## 2. Methods

### 2.1. Burn Injury

Animal experiments were conducted under a protocol approved by MGH Subcommittee on Research Animal Care (IACUC) and were in concordance with NIH guidelines. Male CD-1 mice (Charles River, 27-28 grams, twelve per group) were anesthetized, shaved on the back, and confined to polycarbonate templates exposing 20% TBSA. The exposed area was immersed in a 90°C water bath for 1, 3, 6, or 9 seconds, followed by resuscitation with 2 mL of saline given intraperitoneally. Sham-treated animals received the same treatment except that room temperature water was used. After injury the mice were returned to their cages for 24 hrs with free access to food and water.

### 2.2. Histology

One day after injury, the burn wounds were examined and skin changes were noted. The skin wounds were excised along with the underlying deep paraspinal muscle. The tissue samples were fixed in 10% formalin overnight, sectioned, inserted into cassettes, processed to paraffin blocks, microtome-sectioned to 6-micron sections and stained with H&E for light microscopy. The stained sections were studied for burn pathology and were also used to measure the depth of the burn.

### 2.3. Production of Thigh Abscess with Bioluminescent Bacteria

Bioluminescent *Proteus mirabilis *(Xen 44) and *Pseudomonas aeruginosa (Xen-4) *(kind gift from Xenogen Corp, Alameda, CA) were generated by transposon mutagenesis using a promoterless, complete lux operon (luxCDABE) derived from Photorhabdus luminescens as described elsewhere [9, 10]. The bacteria were grown up overnight and then pelleted, resuspended in fresh media, and diluted until an optical density of 0.3 had been achieved, which in our laboratory has been found to correspond to ~10^6^ microorganisms/mL. Approximately five million bacteria in 0.1 mL of saline were injected (subcutaneous) into the thigh of burned or sham-treated animals at two hours after injury.

In one set of experiments burned mice were treated with ciprofloxacin (5 mg/kg) injected into the thigh with the bacteria or intraperitoneally at the same time that the bacteria were injected in the thigh.

### 2.4. Bioluminescence Imaging

Bioluminescence imaging was performed at various times (3 hours to 20 days after infection) depending on the experimental protocol. Mice were lightly anesthetized using isoflurane and placed in the imaging chamber fitted with a low-light imaging charge-coupled device (CCD) (Hamamatsu Photonics KK, Bridgewater, NJ) as described elsewhere [11]. All images were presented at the same signal bit range to ensure that the bioluminescence signals could be compared.

### 2.5. Statistical Analysis

Survival data were analyzed by the log-rank (Mantel-Cox) method. 

## 3. Results

### 3.1. Macroscopic Findings

Neither the sham controls nor the mice with 1-second burns showed any gross macroscopic abnormalities. The mice with 3-second burns showed mild erythema and a triangular focus of dark red discoloration. The mice with 6-second burns showed mild erythema and swelling, patchy erythema of the deep muscle, and skin-muscle adhesion. The mice with 9-second burns showed swelling, dark discoloration with a prominent oval margin, and large areas of skin firmly attached to the deep muscle.

### 3.2. Histopathological Findings

**Figure 1:**
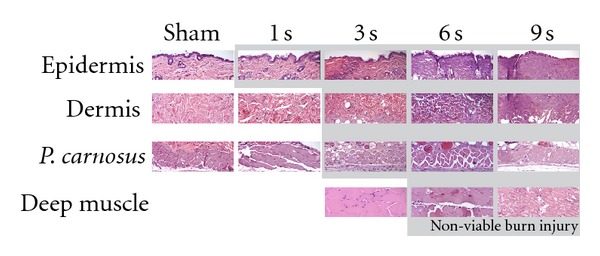
Histology of burn wounds with different exposure times. Tissues were prepared from normal sham-treated skin or from burns following various exposure times to 90°C water.

Figure 1 shows representative H&E sections of sham-treated mice and mice with 1, 3, 6, or 9-second burns. The mice with 1-second burn showed necrosis, confined to the epidermis. The mice with 3-second burn showed injury extending into the panniculus carnosus. The mice with 6-second burn showed deep injury extending into the superficial deep muscle. This deep injury was also seen in the mice with 9-second burn, but there was also significant injury to the deep muscle.

**Figure 2:**
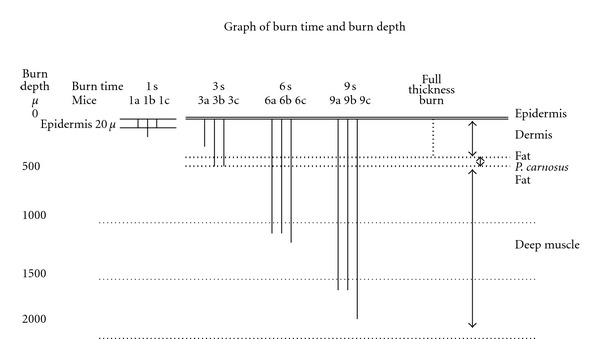
Effect of varying exposure times on burn depth. The depth of burn was determined from tissue histology analyzed microscopically. The letters a, b, and c represent histology from three different mice.

Figure 2 shows a graph of the calculated burn depth versus the time of burn injury for the different time of burn exposure. The 1-second burns were confined to the epidermis (20 micron). The damage in the 3-second burn extended to approximately 500 microns. The damage seen in the 6- and 9-second burns extended 1000 to 1500 microns deep into the skeletal muscle.

### 3.3. Effect of Bacterial Infection on 24 hr Survival

**Figure 3:**
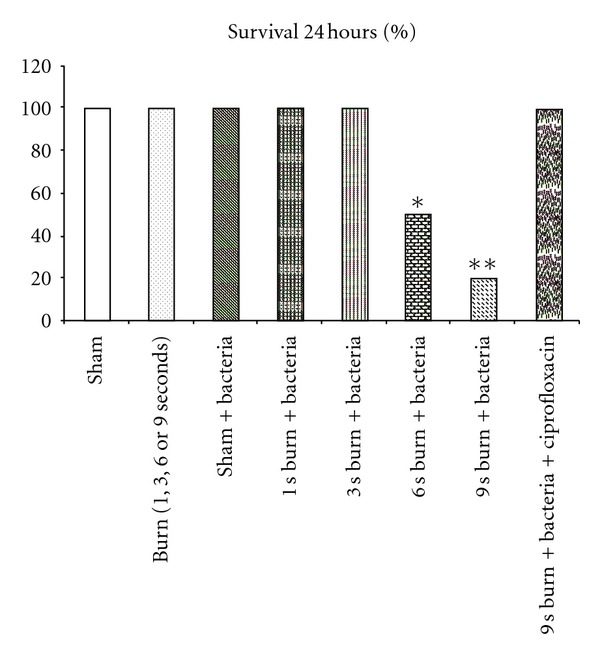
Survival curve of mice with burns with and without bacterial injections. Groups of 12 mice were subjected to sham, burn, or thigh infection as described Section 2. The 24-hour survival was recorded for each group. *P < 0.001 6-second plus infection and 9-second burn plus infection versus all other groups, **P < 0.01 6-second plus infection versus 9-second burn plus infection.

There were no deaths after 24 hrs in the sham-treated mice, sham mice injected in the leg with either *P. mirabilis *or *P. aeruginosa*, mice subjected to 1-second, 3-second, 6-second, or 9-second burns without infection, or mice subjected to 1-second and 3-second burns and injected in the leg with either *P. mirabilis *or *P. aeruginosa*. However, mortality increased when mice with 6-second or 9-second burns were injected in the thigh with either *P. mirabilis *or *P. aeruginosa *bacteria with 50% or more of the mice succumbing after 24 hrs in the mice with 6-second burns and 80% in the mice with 9-second burns (Figure 3). These results for both of the 6-second and 9-second burn with infected groups were statistically significant (*P* < 0.001) by the Log-Rank (Mantel-Cox) method as compared to the other groups. The results for the 6-second burns with infection were statistically significant from the 9-second burn with infection (*P* < 0.01). Ciprofloxacin, injected either in the leg with the bacterial injection, or into the burn eschar itself at the time of bacterial injection, decreased the mortality of the mice subjected to the 9-second burn and bacterial inoculation to zero (Figure 3).

### 3.4. Bioluminescence Imaging In Vivo

**Figure 4:**
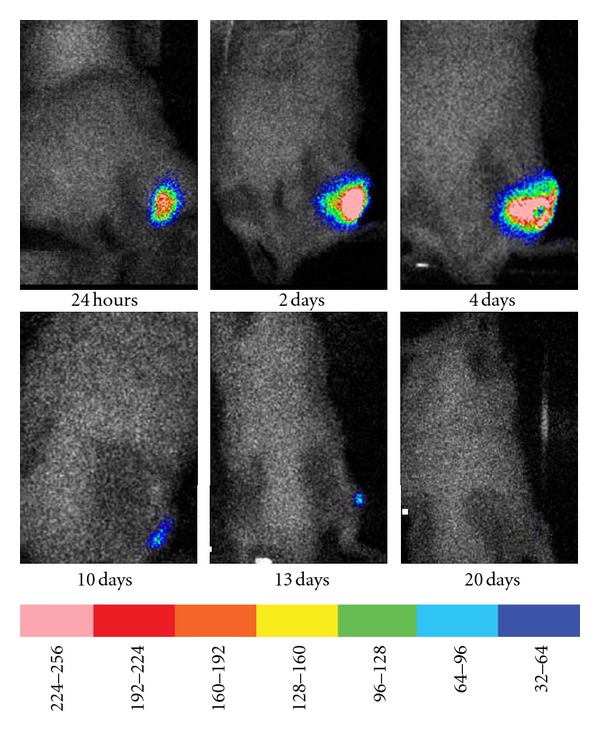
Time course of *P. mirabilis *growth in a sham-treated mouse. Representative time course showing the typical bioluminescence signal that developed in the leg of a sham-treated mouse over the course of 20 days. The scale of pseudocolor indicates the intensity of the bioluminescence, with the higher numbers and pink color corresponding to the highest level of bioluminescence and the lowest numbers and purple color corresponding to the lowest level of bioluminescence.

Figure 4 demonstrates a typical result produced by injection of *P. mirabilis *into the thigh of a sham-treated animal. As can be seen there was a distinct bioluminescence signal visible at the site of injection at 24 hours after inoculation. This bioluminescence continued to increase reaching a maximum at 4 days after-infection. Subsequently, the bioluminescence gradually decreased and was completely absent at 20 days after inoculation. Similar results were obtained in animals injected with *P. aeruginosa *(data not shown). At no time was any bioluminescence observed in the shaven dorsum.

**Figure 5:**
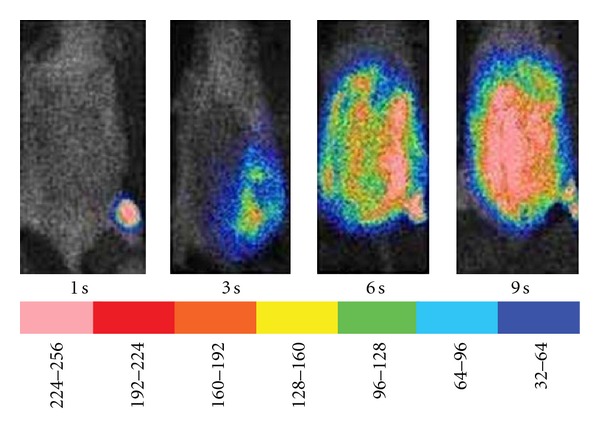
Effect of varying burn depth on infection by *P. mirabilis. *Mice were subjected to 1-second (superficial), 3-second (partial thickness), 6-second (full thickness), or 9-second (full thickness with underlying muscle damage) exposure to the 90°C water, followed by injection of bioluminescent *P. mirabilis* in the thigh and imaged 24 hrs later. The scale of pseudocolor indicates the intensity of the bioluminescence, with the higher numbers and pink color corresponding to the highest level of bioluminescence and the lowest numbers and purple color corresponding to the lowest level of bioluminescence.

Figure 5 shows the results of *P. mirabilis *or* P. aeruginosa *injection into the thigh of burned mice 24 hrs previously. The 1- and 3-second burns did not result in bioluminescence in the burn eschar, but the 3-second burn led to a greater bioluminescence signal in the thigh abscess than was seen with the 1-second burn. Exposure of the skin to 6- or 9-second burns showed an increased bioluminescence in the burn eschar with the signal from the 9-second burn being stronger than that seen with the 6-second burn. Similar results were obtained with *P. aeruginosa *(data not shown). 

**Figure 6:**
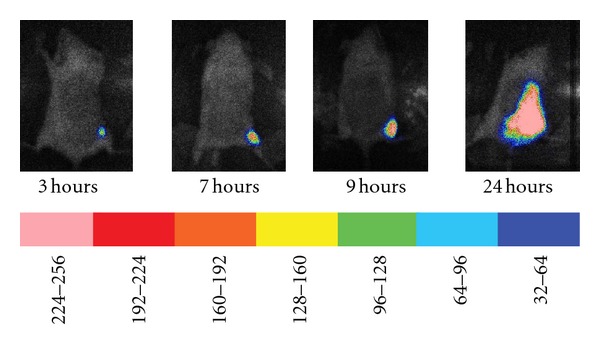
Time course of bioluminescence in a mouse subjected to 9-second burn followed by injection of *P. aeruginosa *in the thigh. Mice were subjected to a 9-second burn, infected in the thigh with *P. aeruginosa* and imaged 3, 7, 9, and 24 hours later. The scale of pseudocolor indicates the intensity of the bioluminescence, with the higher numbers and pink color corresponding to the highest level of bioluminescence and the lowest numbers and purple color corresponding to the lowest level of bioluminescence.

**Figure 7:**
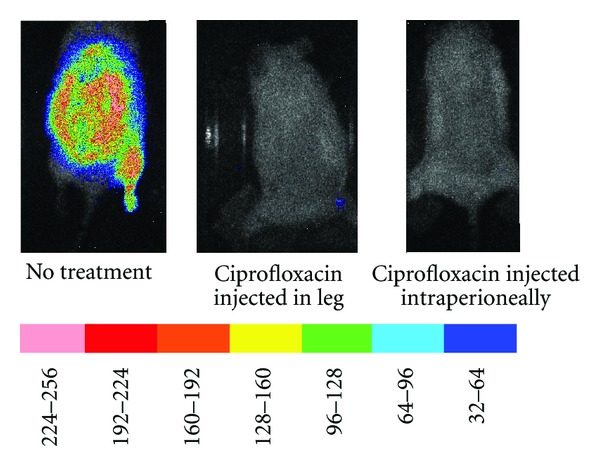
Effect of ciprofloxacin on bioluminescence development in the eschar of mice subjected to 9-second burn at 90°C followed by injection of *Proteus mirabilis* in the thigh. Three mice were subjected to 9-second burn at 90°C and injection of bioluminescent bacteria in the thigh. One mouse had ciprofloxacin injected into the abscess while a second mouse had the ciprofloxacin injected intraperioneally. The scale of pseudocolor indicates the intensity of the bioluminescence, with the higher numbers and pink color corresponding to the highest level of bioluminescence and the lowest numbers and purple color corresponding to the lowest level of bioluminescence.

As illustrated in Figure 6, there was a marked increase in bioluminescence signal in mice that received a 9-second burn followed by injection of *P. aeruginosa *in the thigh over the period from 3 to 24 hrs. The signal in the thigh increased in intensity at 7 and 9 hours following-burn, and at 24 hours following-burn the signal extended to the dorsal burn eschar. Similar results were observed in animals injected with *P. mirabilis *(data not shown). Treatment of the 9-second burned mice infected with *P. mirabilis *or* P. mirabilis *with ciprofloxacin, injected into the leg with the bacteria or intraperitoneally, prevented the development of intense bioluminescence signal in the eschar. The results with three 9-second burned mice infected with *P. mirabilis* in the thigh, two being treated with ciprofloxacin, are shown in Figure 7.

## 4. Discussion

Sepsis remains the major factor resulting in increased mortality and morbidity in burned patients. The burn wound provides an excellent medium for bacterial colonization, in part because there is a reduction in the host immune response produced by the burn injury and in part because of the presence of denatured blood elements and dead tissue to support bacterial growth. The source of the microorganisms producing bacterial colonization in the wound leading to sepsis has been examined extensively and includes the skin, lungs, intestines, and the environment (unpublished results from our laboratory).

 Studies with animal models have been performed to determine if gut bacteria are able to translocate to the mesenteric lymph nodes (MLNs) and beyond, thereby providing a potential source of bacterial colonization of burn wounds [12, 13]. These studies demonstrated that intestinal bacteria can translocate to the MLN after burn injury. However, contamination of the burn wound by enteric bacteria could also be the result of contact with fecal material on bed sheets or dressings in the case of burn patients or bedding material in animal models. It was not clear from these studies with radiolabelled microorganisms if the bacteria that translocated were still alive and growing or simply sequestered in the MLN after translocation.

 In the present study we evaluated whether bacteria growing in an uninjured leg abscess distal to a burn wound can result in burn wound colonization. For this study we used bioluminescent bacteria to be certain that the microorganisms colonizing the burn wound came from the leg abscess. The advantage of bioluminescent microorganisms is that numerous studies have shown that the in vitro or in vivo bioluminescence given off by these bacteria is directly related to the number of microorganisms present.

Our present data demonstrate that as the burn wound becomes deeper, the likelihood of bacterial colonization of the wound increases. We also demonstrated that the bacteria colonizing the wound were alive and growing in the wound based on the increasing bioluminescence over time in vivo. Since the bioluminescent organisms used here have been genetically engineered to produce both bacterial luciferase and its substrate decanal, an increased bioluminescent signal means increased bacterial growth in the tissue, a fact we confirmed (Hamrahi, unpublished observations).

 Our present histological results suggest that varying the times of exposure of the dorsal skin to the 90°C water produced increasing levels of injury with the 9-second exposure producing the greatest depth of injury to the underlying muscle layer. Presumably the heat caused a denaturation of the matrix and a complete disorganization of the tissue resulting in increased leakage of blood borne material into the wound bed.

In this study we observed that the 6-second burns resulted in 50% death and 9-second burns in 80% death with both species *Proteus- mirabilis-* and *Pseudomonas-aeruginosa*-infected mice. It is believed that the virulence of *P. mirabilis *and *P. aeruginosa *is comparable but mediated by somewhat different mechanisms. For *P. mirabilis* it appears that its remarkable motility or “swarming ability” allows it to penetrate through the tissue to reach the blood stream. For *P. aeruginosa* its remarkable expression of an array of tissue destructive enzymes (proteases and lipases) allows it to reach the bloodstream by forging a path through the tissue rather than swimming through the existing gaps.

We did not compare the area of bioluminescence at different time points between animals that survived until 24 h and animals that died before to determine if there was a correlation between early dissemination and death. The reason was in order to measure the bioluminescence, the animals had to be anesthetized. With the very sick burned animals, the additional anesthesia would have lead to increased mortality. Hence, we did not do a study to determine if there was a correlation of bioluminescence of bacteria and early death in the burned mice.

The reasons for the increased colonization of the burn eschar by the bacteria from the abscess in the uninjured thigh may be related to changes in tissue permeability, suppressed immune function, or both. The skin contains antimicrobial peptides that are expressed in keratinocytes, sweat duct epithelia, cells of hair roots and hair bulbs, and vascular endothelium as well deeper portions of the skin [8]. Decreased levels of antimicrobial peptides have been reported to be associated with burns [14]. In this study, colonization of the burn eschar by the bioluminescent bacteria was related to the depth of the burn injury. 

Other cell types, including dendritic cells, may also be involved. Immunopathology will be applied in future studies to investigate the mechanism(s) of this immunosuppression.

 It has also been shown that the permeability of mouse skin increases with the scalding injury [7]. We have examined permeability in both the vasculature and tissue produced by burn injury using L-^18^F-glucose [15], which is not transported into cells but distributes according to simple diffusion. Hence, a change in permeability will produce increased accumulation of L-^18^F-glucose. The dorsal burn injury to mice used in this study (90°C, 9 seconds) resulted in significantly higher accumulations of L-^18^F-glucose compared to uninjured surrounding skin, suggesting increased permeability.

 We did not examine the blood of the burned animals with the various degrees of burn injury after injection of bioluminescent bacteria into the leg to determine if there were more bioluminescent bacteria present in the blood that might have contributed to the increased mortality. However, when the peritoneum of the mice with the 9-second burn was opened, the internal organs (liver and spleen) had a high level of bioluminescence which was not observed in the sham animals or the mice with lesser degrees of burn injury (data not shown). In addition, the antibiotic ciprofloxacin prevented the growth of the bioluminescent bacteria and increased survival. Hence, it is possible to hypothesize that the increased bacterial colonization of the 9-second burn wound eschar is related, at least in part, to bioluminescent bacteria growing in the leg abscess that then gained access to the blood and spread around the body.

 In summary, the present data suggest that bacterial colonization of the burn wound can occur as a result of migration of bacteria growing in an abscess distal to and separate from the burn wound. The eschar colonization increases with increasing burn depth. This model may prove useful in studying the factor(s) relating to burn-induced immunosuppression, wound bacterial colonization, and the severity of burn injury. In addition, this model may allow for further understanding of the molecular mechanisms for the prevention and treatment of burn infections and burn sepsis.
